# Supplementary material for: Are carbon emissions trading and green financial instruments synergistic? -Comprehensive quantitative research based on content analysis
Source: PLoS One. 2024 Mar 7;19(3):e0298601. doi: 10.1371/journal.pone.0298601 (PMC10919601; doi:10.1371/journal.pone.0298601)
Supplement: S1 Data — (ZIP) [file pone.0298601.s001.zip › Data/地级市Z_F工作报告.zip!/╔╜╢1⁄2/╝├─■╩╨/└┤╫╘╣1⁄2╓┌║┼í╛┬φ┐╦╩2╛▌═°í┐_2008.docx]

各位代表：

　　现在，我代表济宁市人民政府向大会报告工作，请予审议，并请市政协委员和其他列席会议的同志提出意见。

　　一、过去五年工作回顾

　　五年来，我们坚持以邓小平理论和“三个代表”重要思想为指导，全面落实科学发展观，团结带领全市人民开拓奋进、扎实苦干，顺利完成本届政府任期目标，“十五”计划提前实现，“十一五”规划深入实施，科学发展、和谐发展迈出坚实步伐。

　　（一）综合经济实力跃上新的台阶。预计2007年全市地区生产总值1730亿元，比2002年翻了一番多、年均递增16.6%；财政总收入突破200亿元，其中地方财政收入超过100亿元、五年增长1.6倍、年均递增20.8%；规模以上固定资产投资五年累计2660亿元，年均增长21.1%；金融机构年末存款余额1223亿元、贷款余额767亿元，比2002年分别增加682亿元和359亿元。我市跨入全国“综合实力百强城市”行列。

　　（二）产业结构层次明显提升。以经济战略转型为主线，纵深推进结构调整，三次产业比例由2002年的17.5：46.8：35.7调整为11.9：55.6：32.5。农业“十大优势特色产业产品”发展规模持续扩大，启动建设96处农产品质量安全示范区，林牧渔业产值占比提高到48%。全市规模以上工业企业发展到2562家、五年新增2012家，实现主营业务收入2200亿元、五年增长3.1倍，利税过千万元企业由77家发展到240家。建成机械制造、生物技术、纺织新材料、专用汽车四大国家级产业基地。五大产业集群增加值对工业经济的贡献份额增加到41%。制造业增加值占规模以上工业的比重达到57.1%。高新技术产业产值占比达到28.5%，五年提高16.7个百分点。累计创出4个中国驰名商标、52个山东省著名商标和100个全省全国名牌产品。鲁南质检中心投入使用。工商、物价监管和统计执法得到加强。相继引入贵和、银座、上海世纪联华、南京中央百货等知名商业品牌，全市社会消费品零售总额586亿元，五年增长1.1倍。我市荣获“中国优秀旅游城市”称号，去年接待中外游客1680万人次，旅游社会收入115亿元，比2002年增长1.9倍。曲阜明故城、嘉祥石雕工业园列为省级文化产业示范基地。包括物流系列项目在内的运河综合开发工程启动。组建起圣泰农村合作银行、济宁市商业银行，成功举办3届银企合作促进会，15家保险企业在我市设立分支机构。民营经济规模实力和贡献份额持续提升，去年上缴税金104亿元，五年增长5.2倍。

　　（三）改革开放实现较大突破。农村综合配套改革取得阶段性成果，农业税、特产税全面取消。国有资产监管体制逐步到位。财政和投融资管理等项改革成效明显。国有粮食购销企业改革全面完成。事业单位改革稳步推进。太阳纸业、如意毛纺成功上市。市属工业困难企业改制脱困取得重大进展。境内外集中招商活动成果丰硕，直接利用外资由2002年的0.88亿美元增加到4.15亿美元，太阳纸业、银河集团等成功走出嫁接外资发展壮大新路子，22家世界500强企业落户济宁。外贸进出口总额增加到28亿美元，其中出口18亿美元、比2002年增长4.1倍。

　　（四）城乡面貌发生显著变化。确立“孔孟之乡、运河之都、水城风貌、生态宜居”城市发展新定位，以中心城区为龙头牵动推进组群结构大城市建设，全市城市化水平由2002年的37%提高到42%。编制完成新一轮城市发展总体规划及一批专业规划。济宁机场正在加紧建设。济菏高速济宁段、崇文大道、滨湖路一期等重点工程相继建成。济宁至北京始发列车定图运行。治淮东调南下、南水北调东线济宁段等重点水利工程进展顺利。完成微山二线船闸建设任务。内河港口吞吐量超过4000万吨，五年翻了一番。首座500千伏高等级输变电站建成运行。中心城区“东拓西跨南联北延”首批12项重点项目深入推进。曲阜孔子文化会展中心、圣都国际会议中心、市文体中心等一批重点工程动工兴建。河湖水系综合整治初见成效。五年新增城市绿地686万平方米，济宁南郊动植物园建成开放，我市荣获省级园林城市称号。城区集中供热面积扩大到692万平方米。天然气引进主体工程竣工。建成12处城市污水处理厂和济宁生活垃圾处理厂。城区管理体制进一步理顺，行政综合执法能力不断加强。新组建的城建投资有限责任公司运营机制逐步完善。经营性用地招拍挂制度执行到位。各县市城市开发建设规模扩大，小城镇建设扎实推进。

　　（五）生态环境质量持续改善。集中落实重点行业重点企业节能减排措施，淘汰83家企业落后产能，成功拆除微山湖煤电公司、华能济宁电厂小火电机组。资源综合利用企业发展到140家，工业“三废”利用率提高到91%。列入国家、省治污规划的重点项目竣工率达到95.3%。建成环保在线监测平台，查处263家违法排污企业。农业面源污染得到有效控制。南四湖人工湿地扩大到5.5万亩。森林覆盖率提高到25%。五年累计治理采煤塌陷地6.5万亩。全市耕地实现占补平衡。境内33家燃煤电厂脱硫改造任务基本完成，秸秆综合利用与禁烧工作得到国务院领导充分肯定，城区空气质量优良率保持在90%以上。预计“十一五”头两年万元GDP能耗降低8.5%，二氧化硫和COD排放量分别削减15.5%、11.9%。去年代表山东省接受国家淮河流域水污染防治检查考核取得好成绩。

　　（六）人民生活水平稳步提高。五年累计新增城镇就业48.47万人，城镇登记失业率控制在4%以内。农村劳动力转移就业112.6万人。全市城市居民人均可支配收入14200元、农民人均纯收入5100元，分别比2002年增长96.5%和71.5%。养老、失业、医疗、工伤保险参保人员增加到312.68万人。7.6万户城乡居民纳入低保范围。五年累计投入社会救助资金6.98亿元。建立城乡困难居民大病医疗救助制度。农村“五保”集中供养率提高到75%。新型农村合作医疗覆盖所有乡镇。完成138个乡镇卫生院改建任务。五年新建改造农村公路9685公里。城乡用电同网同价政策执行到位。3400多个行政村、400多万农民用上自来水。2130个行政村完成“一池三改”。累计改造中小学校舍危房76.7万平方米，城乡义务教育“两免一补”政策落实到位，全市农村教师工资实现县级统筹发放。顺利完成助残济困“三个一千”工程。为20万库区移民落实了后期扶持政策。市政府连年承诺为民所办10件实事和去年为农民群众解决的15个方面问题如期兑现，城乡百姓得到更多实惠。

　　（七）各项社会事业长足进步。15个单位被评为“全国文明单位”，我市荣获全省“创建文明城市先进集体”称号。连年成功举办中国专利高新技术产品博览会及“院士济宁行”活动。五年取得118项省级以上科技进步奖。济宁进入“国家知识产权试点城市”行列。教育优先发展战略深入实施，基础教育切实加强，中等职业学校布局调整基本完成，山东省贸易职工大学新校区启动建设。济宁学院申办成功。由我市倡议设立的联合国教科文组织“孔子教育奖”连续两届在曲阜颁发。一年一度的国际孔子文化节去年首度实现升格举办，影响力、知名度进一步提升。中华文化标志城规划建设得到中央、省高度重视，前期工作加快推进。一批文艺精品在全国、全省获奖。公共卫生“两大体系”建设顺利完成。食品药品监管不断加强。人口自然增长率稳定在5‰左右。县乡全民健身设施日益完善。国防动员、民兵预备役、人民防空（民防）和国家安全工作成效明显。荣获全国双拥模范城“五连冠”。成功举办2007国际慈善论坛。广播电视、新闻出版、档案史志、民族宗教、外事侨务、地震气象及妇女儿童、老龄、残疾人等项事业取得新成绩。夺取抗击非典、抗洪抢险和防控禽流感斗争胜利，保障了人民群众生命财产安全。

　　（八）民主法制建设不断加强。及时向人大报告工作、向政协通报情况，认真贯彻《行政许可法》和国务院《全面推进依法行政实施纲要》，先后精简809个行政审批事项，创新完善市县两级行政审批中心运作机制，依法行政水平不断提高。持续开展“双评”活动，高绩效机关创建扎实有效。全面推行政务公开、村务公开、厂务公开。第七、八届村民委员会换届直选顺利完成。开通“12345”市长公开电话。监察、审计工作力度加大，教育、制度、监督并重的惩治和预防腐败体系逐步健全，源头治腐措施深入落实，治理商业贿赂成效显著，解决了一些损害群众切身利益的实际问题。深入推进“平安济宁”建设，持续开展严打整治斗争，治安防控网络更加严密。切实加强社会应急管理，处置突发事件能力提高。群众工作新机制初步建立，服务平台功能日趋完善。普法、依法治理工作不断深入。矛盾纠纷排查调处措施到位，信访案件责任制有效落实。联手创建鲁苏和谐稳定边界经验做法在全国推广。安全生产形势稳定好转，主要控制指标持续下降。

　　各位代表，过去五年的成就来之不易，归功于省委、省政府和市委的正确领导，得益于历届领导班子奠定的良好基础，离不开市人大、市政协的监督支持，凝结着全市广大干部群众的心血汗水。在此，我代表市人民政府，向全市广大工人、农民、知识分子和干部，向各民主党派、工商联、人民团体和各界人士，向中国人民解放军驻济部队指战员、武警官兵和公安干警，向中央、省驻济单位，向来济宁创业发展的投资者和建设者，向关心支持济宁现代化建设的港澳台同胞、海外侨胞和国际友人，表示衷心感谢和崇高敬意！

　　五年的探索实践使我们深切体会到，要把济宁的事情办好，不断开创改革发展新局面，必须以解放思想为先导，着眼解决“三低三小”问题，深化“三学三创”活动，善于以新理念、新思路、新办法谋划发展方略、破解瓶颈制约，在应对挑战中拓展新路，在抢抓机遇中奋力争先。必须以科学发展观为统领，准确把握市情及发展阶段性特征，加快实现工作指导转变，着力解决影响科学发展的关键问题，自觉贯彻好字优先、好中求快的要求，推动科学发展观在济宁具体化。必须以经济战略转型为主线，加快转变经济发展方式，大力推进结构调整，提高自主创新能力，构建现代产业体系，在转型中打造新亮点、增创新优势。必须以实施项目带动为抓手，把项目作为引领经济社会发展的突破口，调动各方力量运作项目，聚集优势资源投入项目，优化软硬环境建设项目，靠项目带动扩张投资规模、促进产业升级、加快城市化进程、繁荣社会事业。必须以改善民生为目标，坚持富民利民优先，全面落实改善民生各项政策措施，让人民群众充分享受改革发展成果。必须以真抓实干为保证，身体力行“一切工作具体化”要求，练就抓落实的过硬本领，保证各项工作措施落到实处、取得实效。这既是过去实践的经验总结，也是今后工作的指导原则，只要继续坚持并不断丰富完善，我们就能够与时俱进、乘势而上，在科学发展的道路上迈出新的步伐、创造新的辉煌！

　　我们也清醒地认识到，全市经济社会发展还存在一些矛盾问题，政府工作也有许多缺点不足。主要是：经济结构性矛盾依然突出，新兴主导产业特别是现代服务业和高新技术产业成长不快；发展方式没有根本转变，节能减排约束性指标完成不理想；县市区发展差距有所拉大，部分县乡财力紧张；城市化相对滞后，中心城区辐射带动功能不强；社会事业发展存在薄弱环节，一些关系群众切身利益的实际问题尚未根本解决；政府职能转变不快，有些干部思想观念、领导能力、工作作风不能完全适应形势任务要求，少数部门和单位责任意识淡薄、办事效率不高，个别干部以权谋私甚至违法犯罪，影响发展环境的现象时有发生。对此我们一定要高度重视，采取切实措施加以解决。

　　二、新一届政府面临的形势任务

　　今后五年是济宁改革发展的关键时期。我们既面临前所未有的机遇，也将遇到新的严峻挑战。以贯彻落实党的十七大精神为标志，我国开启了全面建设更高水平小康社会的新征程，继续解放思想、坚持改革开放、推进科学发展、促进社会和谐的一系列政策方略更加完善，为我们“四位一体”推进现代化建设指明了前进方向。经济全球化的深入发展，我国主体功能区的规划建设和区域发展布局的战略调整，将促进生产要素大跨度加速流动，为我们依托比较优势、吸纳承接国内外产业资本转移创造了更多有利条件。鲁南经济带规划建设全面启动，一批重大基础设施项目陆续展开，中华文化标志城规划建设加紧推进，孔子文化品牌带动战略深入实施，升格举办后的国际孔子文化节综合带动效应进一步增强，为我们打造优势产业集群、促进新兴产业特别是文化产业大发展、加速经济战略转型带来了难得机遇。我市改革发展的总体思路更加清晰、工作重点更加突出，换届调整后的各级领导班子执政能力不断提高，广大干部群众创业创新的潜能正在源源释放，为我们在更高起点上推进又好又快发展增添了新的动力。同时还要看到，世界经济发展中的不确定因素增多，国家加强改善宏观调控的刚性约束增强，周边地区发展势头迅猛、竞争压力加剧。我们务必增强机遇意识、责任意识和忧患意识，以宽阔的视野审时度势，以争先的姿态高点定位，以开拓的精神克难攻坚，善于顺势而为，勇于自加压力，敢于改革创新，牢牢把握科学发展、和谐发展、率先发展的主动权，朝着全面建设小康社会目标阔步前进！

　　做好今后五年政府工作，必须高举中国特色社会主义伟大旗帜，以邓小平理论和“三个代表”重要思想为指导，认真贯彻党的十七大精神，全面落实科学发展观，加快工作指导转变，坚持好字优先、稳中求进的方针，按照市十一次党代会既定部署，把握“坚持一个总体要求、推进三个率先、实现一个显著提升”的目标定位，围绕经济战略转型这条主线，更加注重调整优化结构、转变经济发展方式，更加注重强化项目带动、提高开放水平，更加注重改革机制体制、推进自主创新，更加注重繁荣先进文化、推动事业进步，更加注重抓好节能减排、建设生态文明，更加注重统筹城乡发展、增强城市承载能力和新农村建设实效，更加注重改善民生、促进社会和谐，努力开创又好又快发展新局面。

　　今后五年的主要任务目标是：

　　——综合经济实力实现新跨越。地区生产总值年均递增14%，力争2012年人均生产总值比2007年翻一番、比2000年翻两番。地方财政收入年均递增16%。全社会固定资产投资年均递增20%。经济战略转型取得更大成效，制造业占规模以上工业比重超过60%，服务业占比提高5个百分点以上，高新技术产业占比达到40%，基本构筑起以高新技术产业为先导，先进制造业和现代服务业为支撑，现代农业为基础的产业格局。

　　——文化繁荣发展实现新跨越。中华文化标志城规划建设取得重大进展，建成一批大型文化基础设施，培育一批特色鲜明的文化品牌，组建一批竞争实力突出的优势文化企业，打造一批省级国家级文化产业基地，初步形成布局合理、功能完善、城乡协调的公共文化服务体系，文化产业占地区生产总值的比重提高到5%，文化综合实力进入全省全国先进行列。

　　——城市化进程实现新跨越。城市特色与风貌进一步彰显，承载能力和品位形象大幅提升，规划建设管理水平显著提高，组团布局的都市区融合发展迈出实质性步伐，中心城区建成区面积超过100平方公里，县城和小城镇面貌明显改观。全市城市化率达到50%以上。

　　——生态文明创建实现新跨越。各类资源实现有序开发合理利用，耕地总量保持动态平衡。循环经济体系初步建立，万元GDP能耗年均降低5%。全面完成南水北调治污规划项目，重点排污企业达标排放，二氧化硫和COD排放量分别削减11.9%、7.8%，力争建成国家卫生城市、国家园林城市和国家环保模范城市。

　　——社会建设实现新跨越。社会管理体系更加健全，政府公共服务职能得到加强。“平安济宁”建设水平不断提升，全社会民主法制观念进一步增强，依法治市进程加快。市民素质和社会文明程度同步提高，争取创建全国文明城市。

　　——人民生活水平实现新跨越。城市居民人均可支配收入和农民人均纯收入年均增长8%。现代国民教育体系初步建立，全面普及高中阶段教育，高等教育毛入学率达到32%。社会就业更加充分，城镇登记失业率控制在4%以内。社会保障体系更加健全，困难群众得到及时救助。公共卫生服务体系不断完善，人人享有基本医疗卫生服务，群众生活的安全感、幸福感明显提高。

　　各位代表，未来五年的发展任务光荣而艰巨。我们要只争朝夕、奋发努力，尽早把济宁建成产业发达、优势凸显、综合实力不断增强的地区，建成城乡秀美、生态良好、人居环境不断改善的地区，建成文化繁荣、事业进步、文明程度不断提升的地区，建成民生殷实、生活富足、全面小康进程不断加快的地区，建成社会和谐、公平公正、安定团结良好局面不断巩固发展的地区，让孔孟之乡焕发出更加蓬勃的生机与活力！

　　三、2008年重点工作

　　今年是新一届政府的开局年，是实施“十一五”规划承上启下的重要一年。我们要按照党的十七大精神和省、市委的部署要求，坚持奋力赶超、跨越发展的基调不动摇，贯彻好字优先、稳中求进的方针不偏离，执行宏观调控、“两个防止”的政策不走样，秉持以人为本、造福百姓的宗旨不懈怠，全力推进又好又快发展。今年全市经济社会发展主要预期目标为：地区生产总值增长15%，地方财政收入增长18%，规模以上固定资产投资增长25%，引进市外国内资金和直接利用外资均增长20%，外贸进出口和出口均增长15%，社会消费品零售总额增长16%，城市居民人均可支配收入和农民人均纯收入均增长8%，城镇登记失业率控制在4%以内。主要约束目标为：万元GDP能耗降低4.7%，二氧化硫和COD排放量分别减少2%、3%，人口自然增长率控制在6.5‰以内。

　　（一）以发展现代农业为首要任务，扎实推进新农村建设。始终把“三农”工作摆在重中之重，突出加强农业基础建设，全面落实强农惠农政策，切实解决农村民生问题，促进农业综合产出效益、农产品质量安全水平和农民群众收入“三个提高”。

　　优质高效农业规模要有新扩展。稳定粮食生产，保障粮食安全，推进国家大型商品粮优质小麦生产基地建设。依托主打品种深度调整农业结构，加快形成区域化布局、规模化发展的特色产业带和聚集区。全年高价值经济作物扩大到450万亩，畜牧业占比提高2个百分点，苗木、花卉及经济林面积新增18万亩，生态、高效、品牌渔业发展到49.5万亩。年内新增规模以上龙头企业100家，发展各类合作经济组织和专业协会500家。

　　农产品质量安全区创建要有新突破。规划建设150处农产品质量安全示范区。推广普及标准化生产实用技术，强化农业投入品施用监管，突出抓好市级农牧渔三大产品检测中心建设。年内力争再有100个无公害、绿色、有机农产品获得省级以上认证。支持帮助各类示范区与大中城市专业市场、连锁超市建立紧密的营销关系，打响“孔孟之乡”优质安全农产品品牌。

　　农业生产条件要有新改善。今冬明春展开1.49万项农田水利建设项目，扎实推进治淮东调南下、南水北调东线济宁段等重大水利工程，高标准实施泗河等骨干河道治理项目，加快中小型水库除险加固进度，健全完善田间排涝灌溉网络体系。鼓励农民购置先进适用的大型农机具。充实加强科技特派员队伍，支持济宁农业高新技术产业示范区争创国家级示范园区。

　　农村公共服务要有新举措。年内新建改造乡村公路1000公里。在600个行政村实施集中供水工程及管网升级改造。推进300个行政村、3万农户实施“一池三改”。全面完成乡镇卫生院改建，年内保证半数以上的村卫生室达到国家标准。改造5万平方米农村中小学校舍危房。实现农村文化信息资源共享工程既定目标，争取60%的行政村通有线电视。年内新增劳动力转移就业10万人，确保每个“零转移农户”至少一人转移就业。深化农村综合配套改革，抓好新型乡镇建设试点。

　　（二）以加快经济战略转型为主线，全面提升优化产业结构。坚持先进制造业与现代服务业“双轮驱动”，推进信息化与工业化深度融合，促使经济结构从资源依赖为主向新兴产业支撑转变，产业层次由初加工、低端化向深加工、品牌化转变，增长动力从投资拉动向创新驱动转变，尽快构建现代产业体系，实现经济战略转型新突破。

　　壮大先进制造业支撑转型。按照“传统产业高新化、新兴产业规模化”的要求，一手抓五大产业集群扩张，一手抓新兴产业培植。一是着力拉长煤化工产业链条。加强境内煤炭企业调控管理，支持兖矿集团、济矿集团以产权为纽带整合资源做大做强。促使国际焦化、国宏化工等优势企业向产业链高端攀升，大力发展低污染、高附加值精细化工和清洁能源产品。加紧建设卡博特碳黑、恒立化工等煤炭精深加工项目。二是提高装备制造业核心竞争力。以实施小松工业园、山推科技园、吉利汽车零部件、重汽商用车二期、倍耐力轮胎和固特异输送带、伊顿液压马达、联诚三期、中集东岳专用车等项目为牵动，扩大10大类终端整机产品规模，带起一批配套协作企业，促使工程机械、重型和专用汽车、内河船舶等主导产业基地和聚集区加速崛起。三是加紧实施纺织服装、医药食品、造纸建材等传统产业改造升级。重点抓好信息化等先进实用技术推广应用，大幅增加技改投入，促其装备水平提高、产品换代升级、整体实力增强。继续实施“优势工业产品膨胀计划”，扩充“梯次强企方阵”。全市新增规模以上企业500家以上，新增利税超千万元企业40家以上，争取太阳、如意、银河三大企业集团销售收入率先突破100亿元。四是扶持电子信息、生物医药、新材料、新能源及节能环保等高新技术产业加速成长。突出抓好光纤光缆、高精密电阻器、谷氨酰胺、基因工程药物、生态纤维、变频调速器等一批骨干产品系列开发和规模扩张，集中扶持新风光电子、正和电子等一批成长性好、发展潜力大的优势企业快速膨胀，壮大高新技术先导示范企业群体。加紧建设中钢多晶硅、三元锂电池等一批技术含量高、附加值高的大项目，一步到位做成高新技术龙头企业。办好济宁软件园。争取高新技术产业投资占比超过15%，高新技术企业新增60家以上，高新技术产值比重提高3个百分点。

　　培育现代服务业助推转型。一是重点发展旅游业。探索建立新型旅游管理体制和运行机制，深度整合开发人文、河湖、生态资源，突出抓好汶上宝相寺二期、曲阜尼山、微山南阳古镇、泗水西侯幽谷、梁山水浒影视基地和北湖度假区等重点项目。着眼文化旅游有机融合，策划推出更多参与性强的文化娱乐项目和高水平旅游演艺活动。培植大型旅游企业集团。建成济宁（曲阜）游客服务中心。积极推行市内旅游景区“一卡通”。结合“吃住行游购娱”系列服务项目开发，加快改造提升传统商贸服务业，大力发展连锁、配送、电子商务等新型流通业态。吸引国内外知名商家进驻建设大型超市和高档酒店。鼓励城市商贸企业向农村延伸拓展经营网络，加快建设农村社区服务中心。加强食品药品监管装备设施和执法能力建设，提高工商、物价监管水平。二是大力发展现代物流业。以实施运河整体开发合资项目为突破口，先期建设两大现代化港口及仓储服务区，同步推进航道改造升级和船舶更新换代，扩大运河物流综合带动效应。依托交通枢纽、通关口岸和空港优势兴建大型物流中心。集中扶持一批骨干物流企业集团。积极兴办第三方物流项目。三是创新发展金融服务业。大力培育各类新型农村金融机构，年内争取新引进1-2家金融企业。完善开放式银企合作平台运行机制，引导各商业银行优化信贷投向，努力提高存贷比。加强金融监管，健全担保体系，扶持发展风险投资。支持更多保险企业丰富品种、拓展市场。四是规范发展房地产业。进一步开放房地产市场，运用多种手段加强房地产市场调控。着力培育一批资质等级高、综合实力强的建安企业集团，引进更多市外大公司和战略投资者参与我市房地产开发。提升建筑设计品位档次，建设高水准的监理机构与队伍。培育完善房地产交易市场，提高房地产业对经济增长贡献度。五是积极发展商务服务业。重点发展律师、公证、会计、审计、资产评估及咨询服务业，不断完善证券、信息、人才、劳动力等市场中介服务业，大力推广代理、代办、经纪、拍卖、担保服务业，逐步形成种类齐全、运作规范的商务服务体系。年内全市服务业投资增幅高出固定资产投资增幅5个百分点，服务业增加值占比提高1-2个百分点。

　　强化自主创新引领转型。加大科技创新投入，发挥企业主体作用，推动产学研结合，加快构建技术创新体系、知识创新体系和创新服务体系。引导企业足额提取研发经费，创建各类技术中心和研发机构，年内省级工程技术研究中心发展到45家，再建成1-2家国家级技术中心。支持重点企业与高等院校、科研院所建立100家产学研合作创新联盟。重点扶持8个重大科技专项和一批引进消化吸收再创新项目。注重利用驻济科技资源加快新技术、新成果就地转化。落实“国家知识产权试点城市”建设各项措施。全年取得重要科技成果160项，专利申请量增长20%。完善鲁南质检中心服务功能，高标准建设国家煤及煤化工产品质量监督检验中心，年内争创5个以上省级、国家级名牌产品。提升中国专利高新技术产品博览会开放度和成果交易量，增强“院士济宁行”活动实效。办好技术市场和市县生产力促进中心。有计划地培养各类实用型、技能型人才，面向海内外优先选聘急需高层次人才特别是领军型创业创新人才。继续实施“百名博士引进工程”。做好引进国外智力工作。重奖贡献突出的科技功臣。

　　创新体制机制保障转型。加大企业上市扶持力度，培植储备后续上市资源，年内力争再有1-2家企业上市融资。努力完成市属困难企业改制脱困任务。实施国有资本经营预算、业绩考核、薪酬分配和财务总监制度，完善国有企业领导人员管理办法。深化非资源型竞争领域国有企业市场取向改革。稳步推进财税、金融和投资体制改革。加快事业单位分类改革。深入落实“促强扶弱”政策措施，支持经济薄弱县加快赶超。持续兴起全民创业热潮，鼓励民间资本兴办二三产业、进入公用事业和基础设施建设等领域，深入实施“中小企业成长计划”，鼓励民营大户走好集团型、科技型、外向型扩张发展之路。

　　（三）以招商引资项目建设为突破口，努力提高对外开放水平。持续展开招商攻势，狠抓项目运作落实，靠扩大开放增加有效投入、增创竞争优势、增强发展后劲。

　　创新招商方式促项目引进。按照“集中活动开路、专业小分队跟进”的模式抓国内境外招商，精心策划组织大型招商活动，扩大日韩、欧美、港台及长三角、珠三角、京津地区招商成果。按照“会前联络、有效对接，会上洽谈、集中签约”的模式抓节会招商，精心组织好国际孔子文化节系列经贸活动。按照政府、企业与外商“三方高层推动”的模式抓企业招商，建立政府领导与优势骨干企业挂钩推进制度，有针对性地与大集团、大商社高层特别是世界500强中国总部开展对接洽谈，力求嫁接外资扩张发展。按照“立足配套成龙、突破上下游项目”的模式抓产业链招商，从主导产业中选择一批专业协作项目，力争在引进相关大公司生产线及配套企业的整体转移上实现突破。坚持招商引资与对外贸易一起抓，配合海关、检验检疫等部门联手推进“大通关”建设，增加机电产品、高新技术产品出口比重。扩大先进技术、关键设备和短缺资源进口。支持有条件的企业到境外建立生产基地、资源基地和营销网络。

　　发挥园区载体功能促项目集聚。统筹省级园区基础设施配套和产业项目引进，实施整体推介招商，增加项目进区密度。制定政策导向明确、利益分配合理的办法措施，引导部门、乡镇和企业招商项目聚集园区。济宁高新区要着力引进技术领先、集中度高、规模效益突出的制造业大项目，争取跨国公司地区总部、研发机构、营销中心入驻。年内省级园区对全市经济增长的贡献份额提高到25%。

　　优化软硬环境促项目落地。深入落实创新发展环境“20条”，组织开展软环境建设集中督查活动。建立项目运作落实“绿色通道”，简化审批程序，提高办事效率。继续实行领导分级包保重点项目制度，严厉打击阻挠破坏重点项目建设的违法行为。加紧规划建设外商集中居住区，切实改善外来投资者工作生活环境。

　　（四）以落实节能减排责任目标为抓手，着力建设生态文明。一是突出抓好重点行业重点企业节能减排。指导督促6个重点行业和100家重点企业分解落实控制目标，集中推广50项新技术新成果，按既定标准和时限抓好重点废水排放企业深度治理，加紧淘汰小火电、小水泥和小化工等落后产能。完成30家企业清洁生产审核，重点培育30家循环经济示范企业和4家示范园区。认真执行新上项目节能评估、环境影响评价和“三同时”制度，严控高耗能项目，严禁重污染项目，坚决防止产业转移中的污染转移。二是加紧推进节能减排重点工程项目。突出抓好燃煤锅炉改造、能量系统优化等重点节能工程。争取列入南水北调东线治污规划的系列项目全面开工。完善城市污水处理厂配套管网，增加除磷脱氮设施。督促各市县加紧建设城市生活垃圾处理厂。积极防治农业面源污染。继续整治大气污染和异味污染。全面推行秸秆综合利用新技术，建立禁烧长效机制。强化各类生态功能保护区建设管理，新增南四湖人工湿地2.5万亩。完成1万亩采煤塌陷地治理任务。年内森林覆盖率提高到26%。三是强化节能减排激励约束。全面推行排污许可证制度，严格执行排污收费标准。强化用能定额管理，在高耗能行业加大差别电价政策实施力度，兑现节能奖励政策。切实加强耕地保护，提高土地节约集约利用水平。足额征收地质环境治理保证金。四是严格节能减排监管执法。定期组织开展专项督查，搞好重点耗能单位节能计量监察和能源审计，强化超标准用能惩戒措施。认真执行节能减排责任目标考核问责制和“一票否决”制度。完善环保在线监测网络，严厉查处偷排偷放和违法排污行为。

　　（五）以做大做强做美中心城区为主导，加大城市建设力度。按照既定城市发展定位和总体思路，协调推进中心城区与县域城镇建设，促进城市空间布局优化、承载能力提升、服务功能完善、特色魅力彰显。

　　建立城市建设提速推进机制。及早完成一批修建性详细规划，超前编制重点区域控制性规划。层层落实城市建设主体责任，强化部门协调联动，保证各个部位和环节工作的高质量、高效率。完善市政府投融资管理中心运行机制，更多地吸引金融机构、大企业和战略投资者参与城市开发建设。依法规范工程招投标，运用市场机制提高拆迁效率、降低拆迁成本。引导建设单位集中优势力量，改进作业方式，确保如期完成建设任务。

　　全力突破重点工程项目。启动建设济徐高速济宁段。规划建设滨湖路二期工程。年内确保济宁机场建成通航。加紧兴建太白楼路东西两座跨河大桥。抓好北湖中路南延改造、济安桥路南延、环城西路贯通等一批路网工程。全面展开西南片区、北湖度假区和城北片区整体开发，力促城南、济北两个新区形成建设规模。新开工12个城中村改造项目。尽早完成人民公园、百花公园升级改造和全民健身广场等项目。同步推进引水进城和古运河南段、西段综合整治开发及氵光 氵府 河北段绿化美化工程。加快建设市文体中心、金融大厦、创新大厦等一批重点工程，确保曲阜孔子文化会展中心、圣都国际会议中心于国际孔子文化节前投入使用。全年新增集中供热面积100万平方米，保证城区15.6万户居民用上清洁安全的天然气。

　　高起点抓好县城和小城镇建设。支持兖州、曲阜、邹城和嘉祥按照都市区一体化布局，以高水准规划为先导，以完善路网为基础，以新城区开发拓展为牵动，以精品工程项目为支撑，加速与中心城区的对接融合。引导其他各县加大旧城改造力度，配套完善基础设施，统筹抓好居民住宅与商贸流通、休闲娱乐、绿化美化及公共服务等项目开发建设。继续推进新农村建设“三二一”示范工程，加快新一轮镇村规划全覆盖，集中建设特色中心镇和一批文明生态村，搞好新型小康农户住宅试点。

　　切实提高城市管理经营效能。强化行政综合执法，坚决查禁影响城市秩序容貌的违法违规行为。增强街道社区自我管理服务功能，探索推行重点区域和部位保洁管理市场化运作机制，全面清除城市出入口、城乡结合部及背街小巷的“脏乱差”死角。严格实行经营性用地招拍挂出让，注重依靠基础设施先期配套和周边环境改善实现土地增值。强化城市公用事业领域招商引资，推动污水和生活垃圾处理等重大基础设施的对外合作经营。

　　（六）以规划建设中华文化标志城为契机，繁荣发展先进文化。发挥文化底蕴深厚、文化资源丰富的独特优势，放大孔子文化品牌带动效应，全面实施孔子文化系列工程，努力提升先进文化引领、服务与创新能力，加快建设文化名市，增强文化软实力。

　　全力推进中华文化标志城规划建设。这是国家的宏大文化建设工程，是山东文化发展的一大盛事与殊荣，也是展示我市“孔孟之乡、东方圣城”独特魅力，打造“中华民族共有精神家园”的重要载体。要把规划建设中华文化标志城作为兴起文化建设新高潮的重大机遇，作为建设文化名市的切入点和重要抓手，按照国家、省部署要求，主动配合上级部门及有关方面充实专门班子，分线展开工作。适时开展全球创意征集，加紧编制高水准的规划设计方案，严格区域规划控制。先期抓好九龙山区生态保护、文物修复及环境综合整治。抓好活动策划和宣传推介，多渠道融通建设资金。建立中华文化标志城与文化、旅游业发展及都市区建设的良性互动机制，增强工程项目的辐射带动效应。

　　创造文化产业发展新优势。从整合文化资源入手，规划建设儒家文化、佛教文化、水浒文化三大文化产业聚集区和运河文化产业带，创建省级国家级文化产业示范基地。吸引市内外各类资本、高层次人才和知名文化企业兴办文化产业项目，推动娱乐、会展、动漫、广告、体育及影视制作等新兴产业加速成长。完成经营性文化单位转企改制任务，组建演艺、报业、出版、广播电视等新型文化企业集团。扩大对外文化交流合作。举全力办好国际孔子文化节、首届世界儒学大会和省第二届文博会。

　　健全完善公共文化服务体系。支持县市区配套建设图书馆、博物馆和群众文化馆，有计划地实施社区、乡镇综合文化站改造和村居文化大院建设。新建更多群众体育设施，组织好奥运圣火传递活动。加紧实施县市有线电视数字化改造。加强网络文化建设管理。深入开展“扫黄打非”斗争。

　　提高“文明济宁”建设水平。深化文明城市、文明单位、文明家庭系列创建活动。扎实推进“诚信济宁”建设。广泛普及科学知识，倡导健康文明生活方式。注重人文关怀和心理疏导，形成互爱互助、平等和谐的良好人际关系。繁荣哲学社会科学。做好第三次全国文物普查工作。加强国防教育、人民防空（民防）和民兵预备役建设，落实优抚安置政策，巩固提高双拥共建成果。重视做好民族宗教、外事侨务和对台工作。统筹解决人口问题，持续稳定低生育水平。

　　（七）以改善民生为目标，推动建设和谐社会。从完善政策、健全制度、增加投入入手，尽心尽力解决关系群众切身利益的实际问题，继续为民办好10件实事，真正让城乡群众生活年年都有新改善，一年更比一年好！

　　优先发展教育，实现“学有所教”。深入推进“双高普九”，创建省级规范化中小学50所，高中阶段升学率提高到85%以上。鼓励县市区创办职教中心。加快建设山东省贸易职工大学新校区。扩大济宁职业技术学院建设规模。整合迁建济宁市技术学院。帮助驻济高校解决建设发展的实际问题。支持济宁学院完善装备设施、提高办学水平。健全以县为主的农村义务教育管理体制，实现县域范围教师工资统一标准发放。对农村义务教育阶段学生免费提供教科书，提高寄宿生生活费补助标准。完善困难家庭学生救助制度，决不让一名学生因家庭贫困而上不了学、上不起学！

　　努力扩大就业，实现“劳有所得”。今年全市城镇新增就业8.5万人、安置下岗失业人员再就业3.8万人。注重引进建设就业容量大的产业项目，发挥好中小企业特别是第三产业吸纳就业的作用。完善就业援助机制和创业帮扶机制，鼓励扶持下岗职工、失业人员、大中专毕业生、复转军人及进城务工人员等不同群体自主创业、自谋职业，实现城镇“零就业”家庭“动态消零”。认真落实《劳动合同法》，适时提高、严格执行最低工资标准，坚决纠正拖欠农民工工资行为。健全劳动、资本、技术、管理等生产要素参与分配的制度，落实鼓励置业等方面的政策措施，不断提高经营性收入和财产性收入在居民收入中的比重，让更多城乡百姓尽快富起来！

　　优化医疗服务，实现“病有所医”。进一步完善城镇职工医疗保险制度，启动实施城镇居民基本医疗保险试点，力争明年所有城镇居民都能享受基本医疗保障。改善乡镇卫生院和村卫生室装备条件与医疗服务水平，两年内把参加新农合农民的财政补助标准由每人每年40元提高到80元。管好用好城乡困难居民大病救助基金。合理配置城市卫生资源，年内全市社区卫生服务人口覆盖率达到80%以上。以大型公立医院为依托，建立城乡医院对口支援、大医院与社区卫生机构双向转诊制度。积极发展中医药事业。强化医德医风建设，推出更多惠民举措，有效解决“看病难”、“看病贵”问题，真正让群众看得起病、看得好病！

　　健全社保体系，实现“老有所养”。进一步扩大养老、失业等基本保险覆盖面。强化城乡低保对象动态管理，建立保障标准随居民收入和物价水平相应增长机制。切实兑现被征地农民基本生活保障政策。健全社会救助体系，发挥慈善机构、红十字会在扶贫济困中的积极作用。强化残疾人等特殊困难群体的扶助救济措施。做好妇女儿童和老龄工作。年内为80%以上的乡镇敬老院配备锅炉和供热设施，保证“五保”老人温暖过冬、安享晚年！

　　改善住房条件，实现“住有所居”。建立合理的住房供给体系，扩大面向中低收入家庭的普通商品住宅供应量，严格控制房价过快增长。县市区全面建立廉租住房制度。年内济宁城区开工建设10万平方米经济适用房，解决1600个“双困户”住房问题。规范住房公积金管理。改造建设困难农民住房7.5万平方米，彻底消除农村土坯房，让更多老百姓安居乐业！

　　完善社会管理、维护社会稳定是各级政府的重大政治责任。要深入开展平安创建活动，健全治安防控体系，加强城乡社区警务工作，抓好重点部位和治安后进地区的集中整治，依法防范打击违法犯罪行为。优化群众工作平台服务功能，强化司法行政基层基础工作，坚持领导定期接访制度，畅通群众利益诉求渠道，及时化解各种矛盾纠纷和不稳定因素。创新鲁苏边界保障和谐稳定长效机制。完善社会应急管理体制，有效应对处置各类突发事件。重视做好国家安全工作。提高气象、地震监测预报水平，增强防灾减灾能力。落实政府监管责任和企业主体责任，毫不放松地抓好隐患排查和专项整治，严格执行事故责任追究制度，促进安全生产形势稳定好转，全力保障人民群众生命财产安全！

　　四、全面加强政府自身建设

　　按照为民、务实、清廉的要求，进一步加强政府机关思想作风建设，切实提高执政能力和领导水平，以奋发有为的精神状态、争先创优的进取意识和求真务实的工作作风，更好地担负起推进又好又快发展、全面建设小康社会的历史重任。

　　（一）坚持勤于学习，建设创新型政府。深入学习党的十七大精神，准确把握中国特色社会主义理论体系本质内涵，用马克思主义中国化最新成果武装头脑、指导实践、推动工作。坚持在大局下行动，坚决贯彻上级决策部署，自觉服从市委统一领导，确保令行禁止、政令畅通。以深化“三学三创”活动为抓手，按照省委“四破四立”的要求，树立科学发展新理念，把握发展阶段新变化，顺应人民群众新期待，在更高境界上解放思想、更新观念，善于把上级部署与济宁实际结合起来，把长远目标与当前任务结合起来，把满腔热情与务实态度结合起来，务求各项决策更加科学合理，政策措施更加切实可行，克难攻坚更加富有成效。

　　（二）坚持务实高效，建设服务型政府。大力倡导科学、精细、务实的工作作风，力戒形式主义、官僚主义，力戒心浮气躁、急功近利，力戒简单粗放、盲目蛮干。对重点任务目标层层分解，逐一落实执行责任，加大督导检查力度，严格考核兑现奖惩。从市政府做起，下决心精简会议、文件，摆脱繁琐事务，不搞没有实际意义的检查、评比、达标活动，带头深入一线、靠前指挥，重点工作事项牵头主抓、亲历亲为，注重到条件差、困难多、工作进展慢、群众不满意的地方解决问题、寻求突破。强化行政效能监察，建立绩效评估制度，查处纠正“三乱”行为，支持保护各类企业和投资主体在济宁放心、放胆地兴业发展！

　　（三）坚持依法行政，建设法治型政府。认真执行人大及其常委会决议决定，积极支持人民政协履行职责，主动听取各民主党派、工商联、无党派人士和人民团体的意见建议，及时办理人大代表建议和政协提案。积极推进行政管理体制改革，加快实现政企分开、政资分开、政事分开和政府与市场中介组织分开。完善群众参与、专家咨询与集体决策相结合的决策机制，提高决策的科学化、民主化水平。落实国务院《政府信息公开条例》，完善新闻发布制度，加强电子政务建设，办好“12345”市长公开电话。全面加强政府法制建设，严格依照法定权限和程序行使职责权力。提高行政执法队伍素质，落实行政执法责任制和责任追究制，进一步精简行政审批事项，创新行政许可、行政处罚管理机制，强化行政复议和执法监督，确保公正、文明执法。

　　（四）坚持严格自律，建设廉洁型政府。自觉强化党性修养，努力改造主观世界，筑牢拒腐防变的思想道德防线。模范遵守领导干部廉洁自律各项规定，自觉践行廉政承诺。全面落实党风廉政建设责任制，加强对领导班子、领导干部的教育、管理和监督。完善源头治腐各项制度规章。深入治理商业贿赂。切实纠正损害群众利益的不正之风。进一步加强审计监督，支持纪检监察机关依法依纪查办案件，以预防惩治腐败的实效、干净干事的形象，让广大人民群众从心里说党和政府好！

　　各位代表！我们正站在新的历史起点上，团结带领干部群众开创跨越发展的宏伟事业。这是时代赋予我们的神圣使命，也是人民对我们的殷切期望。让我们以深入贯彻党的十七大精神为动力，在中共济宁市委的坚强领导下，坚定信心、振奋精神，同心同德、团结实干，为开创济宁的美好明天，夺取全面建设小康社会新胜利而努力奋斗！
